# Down‐regulation of SLC35C1 induces colon cancer through over‐activating Wnt pathway

**DOI:** 10.1111/jcmm.14969

**Published:** 2020-01-21

**Authors:** Minzi Deng, Zhihong Chen, Jieqiong Tan, Heli Liu

**Affiliations:** ^1^ Department of Gastroenterology The Third Xiangya Hospital of Central South University Changsha China; ^2^ Hunan Key Laboratory of Nonresolving Inflammation and Cancer Changsha China; ^3^ Department of Pathology The People's Hospital of Hunan Province Changsha China; ^4^ Center for Medical Genetics School of Life Sciences Central South University Changsha China; ^5^ Department of Gastrointestinal Surgery Xiangya Hospital of Central South University Changsha China

**Keywords:** colon cancer, fucosylation, SLC35C1, Wnt signalling, β‐catenin

## Abstract

The canonical Wnt signalling pathway is a critical pathway involved in the proliferation of cells. It has been well‐established that it plays the central role during colorectal carcinogenesis and development. Yet the exact molecular mechanism of how the canonical Wnt pathway is fine‐tuned remains elusive. We found that SLC35C1, a GDP‐fucose transporter, negatively regulates the Wnt signalling pathway. We show here that SLC35C1 is reduced in all colon cancer by both immunohistochemistry images and TCGA data, whereas β‐catenin is increased. Down‐regulation of SLC35C1 is also detected by real‐time PCR in stage 3 and stage 4 colorectal cancer tissues. Moreover, analysing the TCGA database with cBioPortal reveals the negative correlation of SLC35C1 mRNA level to the expression of β‐catenin. Reduced SLC35C1 significantly promotes cell proliferation and colony formation of HEK293 cells. Meanwhile, in HEK293 cells silencing SLC35C1 activates canonical Wnt pathway, whereas overexpressing SLC35C1 inhibits it. Consistently, the reduction of SLC35C1 in HEK293 cells also elevated the mRNA level of Wnt target genes *C‐myc*, *Axin2* and *Cyclin D1*, as well as the secretion of Wnt3a. In conclusion, we identified SLC35C1 as a negative regulator of the Wnt signalling pathway in colon cancer. Decreased SLC35C1 may cause over‐activation of Wnt signalling in colorectal cancer.

## 1 INTRODUCTION

Colon cancer, accounting for 8% of new cancer cases, is the third most common type of cancer diagnosed in the United States.^1^ Most colon cancer cases begin as benign polyps and then develop into uncontrolled cell growth. Wnt signalling pathway, a highly evolutionary conserved pathway that modulates cell fate determination, proliferation, migration and behaviour, is known to be implicated in a variety of cancers and other diseases.^2^, ^3^ Wnt signalling pathways are usually divided into the canonical pathway that is β‐catenin (encoded by CTNNB1)‐dependent, and the β‐catenin‐independent non‐canonical pathway, also known as planar cell polarity or Wnt/calcium pathway. It is well established that the canonical Wnt pathway plays a critical role in both physiology and pathology progress of the human adult intestine. In the canonical Wnt pathway, the signal is passed on by the binding of secreted Wnt ligands to their receptors on the cell membrane,^4^, ^5^, ^6^ which subsequently causes the removal of axin from the β‐catenin destruction complex via phosphorylation of the dishevelled protein.^7^, ^8^, ^9^, ^10^, ^11^ This event then leads to the stabilization and accumulation of β‐catenin in the cytoplasm and its eventual translocation into cell nuclear, where β‐catenin activates the transcription factors of TCF/LEF family and starts the transcription of various downstream genes,^12^ such as C‐myc,^13^ Axin2^14^ and survivin.^15^ Over‐activation of Wnt signalling is frequently observed in colon cancer, in particular, the loss of Wnt negative regulator via mutation of the adenomatous polyposis coli gene (APC).^16^, ^17^, ^18^ However, the exact mechanism of aberrant Wnt activation remains largely unexplained.

SLC35C1, also known as GDP‐fucose transporter 1, CDG2C or FUCT1, is a member of the solute carrier (SLC) group protein. It is first cloned from leucocyte adhesion deficiency II (LAD II) patients, who showed reduced GDP‐fucose transported into Golgi.^19^ In the current study, we aim to discover the mechanism of how SLC35C1 regulates the canonical Wnt pathway in colon cancer. Here we show that SLC35C1 mainly locates in secretion‐related subcellular structures, including the Golgi apparatus, ER, and early and late endosomes by immunofluorescence. Immunohistochemistry images of colon cancer tissues and study of the TCGA (The Cancer Genome Atlas) database both implied the reduction of SLC35C1 and elevation of β‐catenin in all stages of colon cancer. To confirm this, we then performed real‐time PCR in the colon cancer tissue and normal tissue. The results showed that the mRNA level of SLC35C1 decreased in stage 3 and stage 4 colorectal cancer. Moreover, we analysed the TCGA database with online tool cBioPortal and discovered that the mRNA level of SLC35C1 negatively correlates with the expression of β‐catenin, indicating the inhibitory effect of SLC35C1 expression on the β‐catenin level. Consistently, cells absent with SLC35C1 showed significantly promoted cell proliferation and colony formation, suggesting the induced Wnt pathway activity by SLC35C1 reduction. This is supported by our next TOPFLASH assay which showed that in HEK293 cells, silencing or overexpressing SLC35C1 causes increased or decreased luciferase signal, respectively. Besides, silencing SLC35C1 in HEK293 cells leads to increased secretion of Wnt3a and Wnt target genes, such as C‐myc, Axin2 and Cyclin‐D1, suggesting activation of the Wnt pathway. Taken together, these results indicate that SLC35C1 is a negative regulator of the canonical Wnt pathway, and loss of SLC35C1 promotes colon cancer progression through the activation of the Wnt signalling pathway.

## 2 MATERIALS AND METHODS

All experiments and methods were performed following relevant guidelines and regulations. All experimental protocols were approved by a named institutional/licensing committee. The use of human tissues was approved by the Ethics Committee of the Third Xiangya Hospital of Central South University, and patients’ consents were obtained.

### 2.1 Expression plasmids and shRNAs

Full‐length SLC35C1 cDNA with a flag tag amplified from a human foetal brain library was cloned into the pcDNA3.1 vector (#v70920, Invitrogen). The LentiCRISPR V2 system was from Addgene (#52961). The TCF reporter plasmid kit containing TOPFlash reporter plasmid and activator plasmid was from EMD Millipore (#17‐285). The Renilla Luciferase control plasmid pRL was from Promega (#E2261). The integrity of all constructs was confirmed by gene sequencing. The shRNA‐SLC35C1 plasmids and control plasmids were from Santa Cruz Biotechnology (#sc‐96300‐SH).

### 2.2 Antibodies

The antibodies we used are as follows: Mouse monoclonal actin antibody (#3700, Cell Signaling); Tom20 Rabbit monoclonal antibody (#42406, Cell Signaling Technology); Rab5 Rabbit monoclonal antibody (#3547, Cell Signaling Technology); Rab7 Rabbit monoclonal antibody (#9367, Cell Signaling Technology); Lamp1 Rabbit monoclonal antibody (#9091, Cell Signaling Technology); Calnexin Rabbit monoclonal antibody (#2679, Cell Signaling Technology); β‐catenin Rabbit monoclonal antibody (#8480, Cell Signaling Technology); M2 mouse monoclonal antibody (#ab60335, Abcam); SLC35C1 rabbit polyclonal antibody (#ab60336, Abcam); Goat anti‐mouse‐Cy3 (115‐165‐146, Jackson ImmunoResearch Laboratories); Goat anti‐rabbit‐Cy2 (111‐225‐144, Jackson ImmunoResearch Laboratories, PA, USA); peroxidase Affinipure Goat Anti‐mouse IgG (115‐035‐003, Jackson ImmunoResearch Laboratories); and peroxidase AffiniPure Goat Anti‐Rabbit IgG (111‐035‐144, Jackson ImmunoResearch Laboratories).

### 2.3 Cell culture, transfection and shRNA

HEK293 cells and L Wnt‐3A cells were cultured in Dulbecco's modified Eagle's medium (#11965, Invitrogen) supplemented with 10% foetal bovine serum (FBS, #10100147, Invitrogen) and 1% penicillin/streptomycin (#10378016, Invitrogen) at 37C, 5% CO2 atmosphere. The shRNA plasmids were obtained from Sigma‐Aldrich. The sequences were as follows:

shRNAslc35c1‐1: CCGGCGGCGTCATCATTGGTGGTTTCTCGAGAAACCACCAATGATGACGCCGTTTTTG

shRNA slc35c1‐2: CCGGGCTCAAACAGACCACTTCCTTCTCGAGAAGGAAGTGGTCTGTTTGAGCTTTTTG

The transfection was performed with Lipofectamine 2000 (#11668019, Invitrogen) according to the manufacturer's instructions.

### 2.4 Western blot

Protein concentration was determined using the BCA protein assay kit (#23225, Invitrogen) prior to immunoblot analysis. Before loading on 12% SDS‐polyacrylamide gel (#PCG2002, Sigma‐Aldrich) for electrophoresis, samples were mixed with SDS sample buffer (#PCG3009, Sigma‐Aldrich) and DTT (#PCG3005, Sigma‐Aldrich) thoroughly. Then, proteins were transferred to a nitrocellulose membrane (#1620115, Bio‐Rad) and the non‐specific binding was blocked by incubating the membrane in PBST (PBS containing 0.05% Tween 20, #P3563, Sigma‐Aldrich) with 5% non‐fat milk for 120 minutes at room temperature. After that, the membrane was incubated with different primary antibodies overnight at 4°C followed by incubation with horse‐radish peroxidase‐linked secondary antibody (Sigma‐Aldrich) in PBST with 5% milk for 1 hour at room temperature. Bands were detected by enhanced chemiluminescence (ECL) reagent (#32106, Invitrogen) and exposed to X‐Omat films (#F1274, Sigma‐Aldrich). To calculate the intensity of bands, we used the ‘Gel analysis’ function of ImageJ software, according to the instruction.

### 2.5 Immunofluorescence

Cells were seeded in 24‐well plates and maintained in DMEM medium containing 10% FBS for 16 hours prior to transfection. The cells were transfected with plasmids using Lipofectamine 2000. After 24 hours, the cells were washed with 1× PBS and fixed with PBS containing 4% w/v paraformaldehyde (#P6148, Sigma‐Aldrich, MO, USA) for 15 minutes. After washing three times, the cells were permeabilized by incubation in 1× PBS containing 1% Triton‐X 100 and 1% bovine serum albumin (BSA, #A1933, Sigma‐Aldrich) for 1 hour and then incubated with the primary antibodies for 2 hours at room temperature. Appropriate secondary antibodies were used to detect the corresponding protein, and the cells were stored in the dark at 4°C until being visualized under laser confocal microscope (Leica).

### 2.6 Fluorescent Immunohistochemistry

Paraffin‐embedded sections were rehydrated by immersing the slides in xylene/ethanol series. Slides were then blocked with 1% horse serum in PBS for 30 minutes at room temperature. After blocking, slides were incubated with diluted primary antibodies at 4°C overnight and then washed three times for 15 minutes each with PBST, followed by incubation with appropriate diluted secondary antibody for 60 minutes at room temperature. Slides were then washed with PBST again, mounted and stored at 4°C until imaging. The staging of the samples is based on the American Joint Committee on Cancer (AJCC) TNM system.

### 2.7 Image process

The Leica TCS SP5 and its supporting software LAS X were used to capture the image. Then, the images were processed with ImageJ. To analyse the correlation between SLC35C1 and β‐catenin, we did a colour profiling assay using the ImageJ ‘RGB Profiler’ plug‐in. Briefly, on the IHF images, a random line was drawn on the SLC35C1 particles of the merged image, and colour profiles of both green and red channels were plotted for the selected line. The line profile implies the distribution of the two fluorophores on the given line. The *X*‐axis represents the distance along the line, and the *Y*‐axis represents the fluorescent intensity.

### 2.8 Real‐time PCR

Real‐time PCR was performed with One‐Step TB Green PrimeScript RT‐PCR Kit II (#RR086, Takara) following the manufacturer's instruction. The primers for C‐myc, Axin2 and Axin2 were described by Leung et al.^20^

Primers for real‐time PCR of SLC35C1:

Forward: AAGATCAAGAGGCCAGCAGA

Reverse: GGAAGGAGCTTGCTTGTTTG

### 2.9 TOPFLASH assay

TOPFLASH assay is an assay usually used for detecting the activity of the canonical Wnt pathway. Briefly, one 24‐well plate of HEK293 cells was transfected with 100 ng of the TOPFLASH reporter plasmid and 10 ng Rellina plasmids pRL per well. Twenty‐four hours after transfection, the cells were stimulated with conditioned medium collected from L Wnt‐3A cells obtained from ATCC (ATCC^®^ CRL‐2647™). Twelve hours later, HEK293 cells were washed once with PBS and then lysed for 15 minutes at room temperature. The lysates were clarified by centrifugation at 20 000 *g* for 10 minutes, and 20 µL of each lysate was used to measure luciferase reporter gene expression (luciferase assay kit, #E1910, Promega). The luciferase activity was normalized to Renilla luciferase activity from co‐transfected plasmid pRL. The relative luciferase activity will increase when the canonical Wnt pathway is activated and decrease if the pathway is inhibited. All experiments were performed in duplicate and repeated at least 3 times.

### 2.10 Cell growth curve analysis

Control HEK293 cells and cells with SLC35C1 silenced or overexpressed were seeded in 6‐well plates. Each well started with 50 000 cells, and 4 time‐points (0, 24, 48 and 72 hours) were calculated. Each time‐points were prepared in triplicates. To calculate cells, wells were trypsinized and cell numbers were counted under a stereomicroscope.

### 2.11 Soft agar colony formation analysis

A total of 5% agarose gel was prepared with RPMI1640 (R0883, Sigma‐Aldrich) complete medium, autoclaved and then used as the bottom layer. HeLa cell suspension mixed with a 5% agar solution was added to the solidified bottom layer and allowed 30 minutes at room temperature to solidify. A top layer of complete medium was added to the wells to prevent drying of agar. Then, plates were maintained in a 37°C humidified incubator with 5% CO2 for 3 weeks. The number of colonies per well was counted with an Oxford Optronics Gelcount Colony Counter (Oxford Optronics). Each condition was performed in triplicates.

### 2.12 TCGA data analysis

Expression of SLC35C1 and β‐catenin, as well as the correlation of their level to the pathologic status of colon cancer, were extracted from the TCGA database (The Cancer Genome Atlas) using the web tool UALCAN.^21^ TPM (transcripts per million) values were employed to estimate the significance of the difference in gene expression levels between groups. The *t* test was performed using a PERL script with the Comprehensive Perl Archive Network (CPAN) module ‘Statistics::TTest’ (http://search.cpan.org/~yunfang/Statistics-TTest-1.1.0/TTest.pm).

The correlation of the SLC35C1 level and β‐catenin was calculated using the TCGA database by cBioPortal (http://www.cbioportal.org). In this work, we analysed the mRNA expression of SLC35C1 and β‐catenin in tumour set containing 382 samples with mRNA data (RNA seq V2) of the TCGA provisional study. mRNA profile is calculated by RNA Seq V2 RSEM and expressed in log2 value.

### 2.13 Statistics

All experiments were repeated three times. The data were presented as mean ± SD and analysed by ImageJ and GraphPad Prism 7 (GraphPad Software). The statistical significance was estimated by unpaired Student's *t* test, one‐way ANOVA or two‐way ANOVA except the statistical results done with online tools, with 0.05 as the level of significance.

## 3 RESULTS

### 3.1 Distribution of SLC35C1

**Figure 1:**
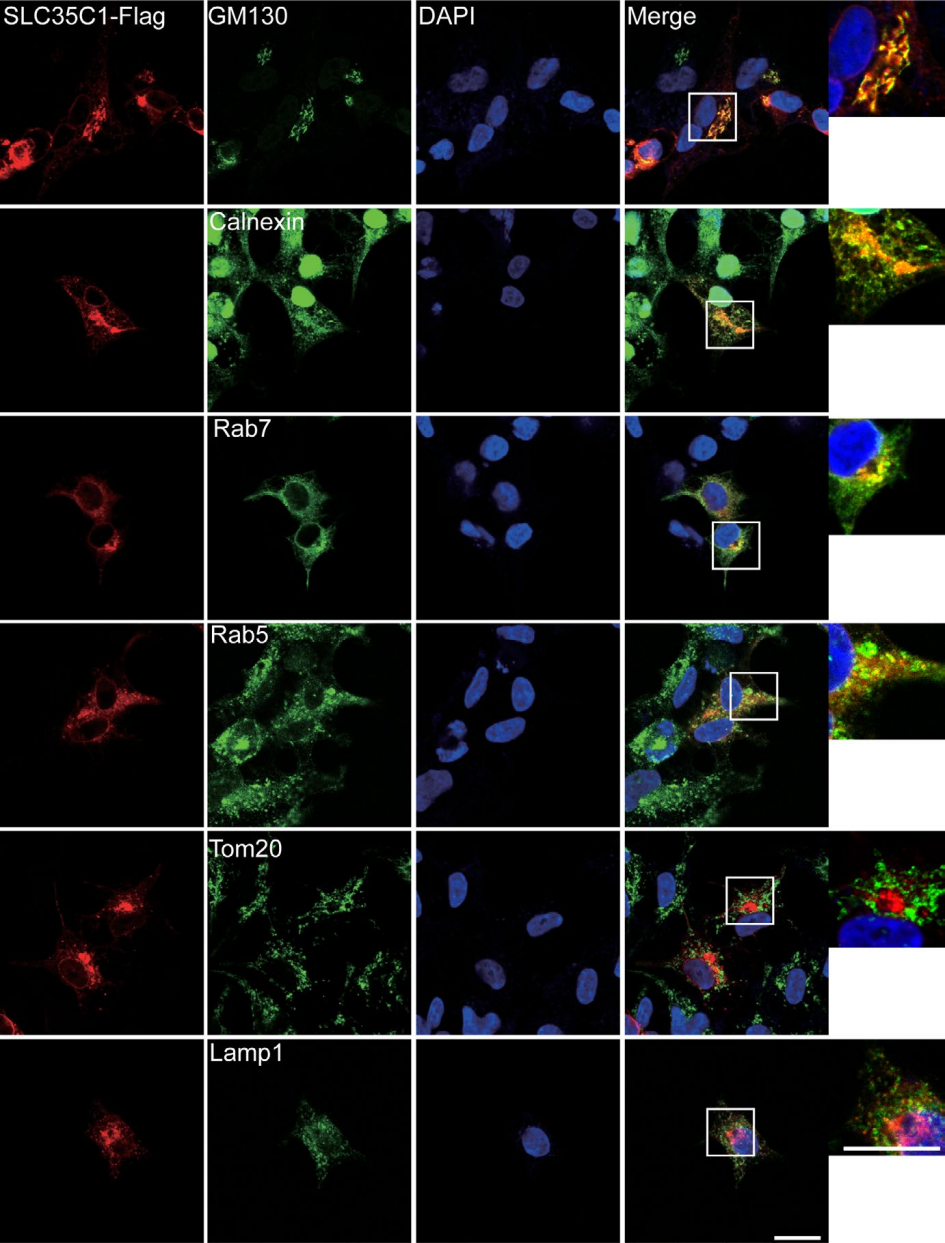
Subcellular distribution of SLC35C1HEK293 cells were transfected with Flag‐tagged SLC35C1. 48 h later, immunofluorescence was performed with antibodies against Flag tag, GM130 (a marker for Golgi apparatus), calnexin (a marker for ER), Rab5 (a marker for early endosome), Rab7 (marker for late endosome), Lamp1 (a marker for lysosome) and TOM20 (a marker for mitochondria). Confocal images demonstrated that SLC35C1 colocalizes with GM130, calnexin, Rab5 and Rab7, indicating SLC35C1 locates at the Golgi apparatus, ER, and early and late endosome. No colocalization SLC35C1 with Lamp1 or TOM20 was found, suggesting SLC35C1 is not in lysosome or mitochondria. Bar = 10 μm

**Figure 2:**
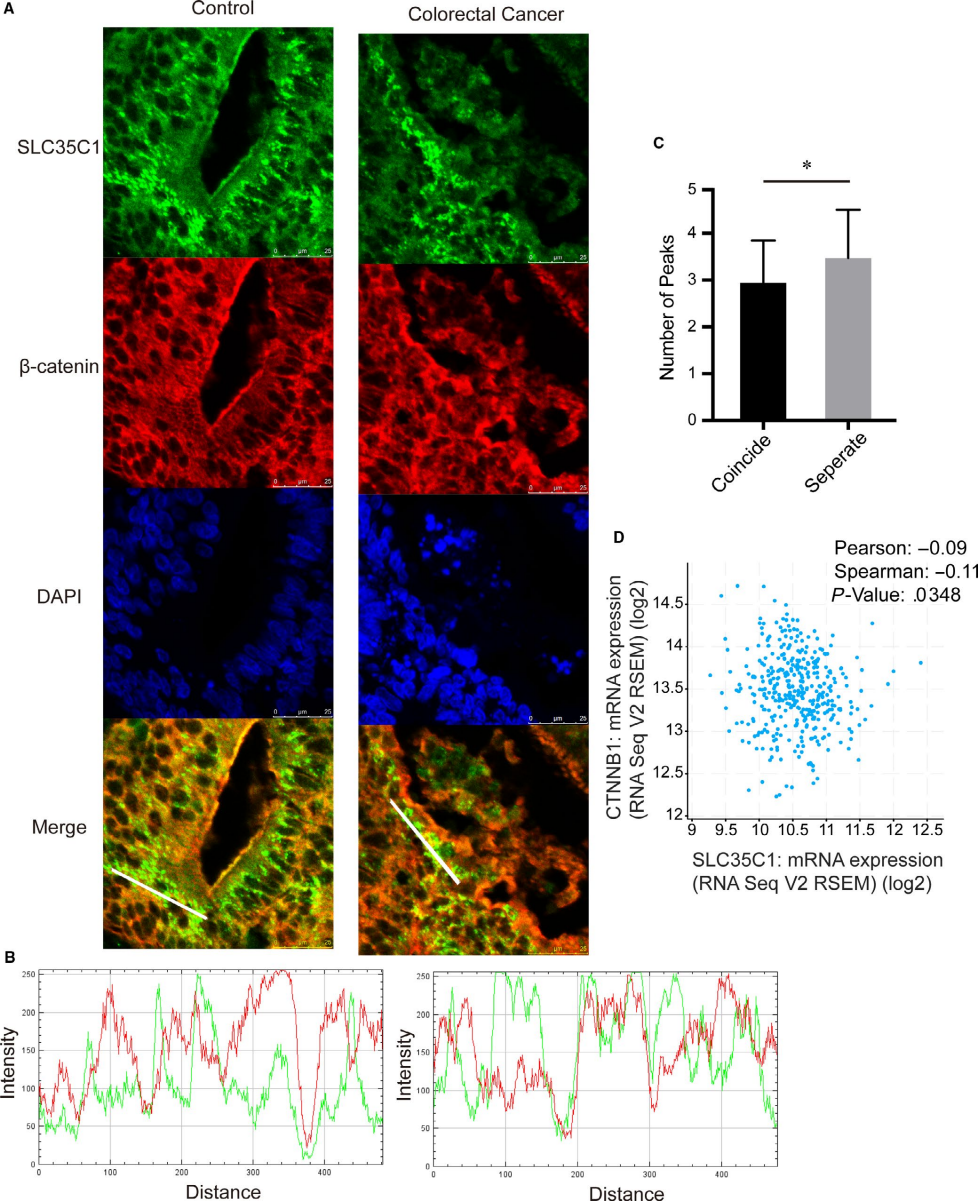
Distribution of SLC35C1 in tissue. A, Tissues from normal donor and stage 2 colon cancer patients were subjected to fluorescent immunohistochemistry with antibodies against β‐catenin (red) and SLC35C1 (green). The images show that β‐catenin does not coincide with SLC35C1 in both normal and cancer tissue. B, The colour profile shows the separated distribution of SLC35C1 and β‐catenin. Multichannel line profile analysis is done by RGB profiler plug‐in of ImageJ. Briefly, a random line is drawn through the green particles on the merged image, and the profile of both green channel and red channel along the selected line is plotted by the RGB profiler, with the X‐axis implies the distance on the line and the Y‐axis implies the fluorescent intensity. C, The intensity of SLC35C1 negatively correlates with that of β‐catenin. Six samples from the control group and 6 samples from the cancer group were included; for each sample, 3 random lines were selected and plotted for colour profiling. Based on the colour profile, the numbers of places where the two‐colour peaks coincide together or separates were counted (Mean ± SD, *P = .02, two‐tailed t test with Welches’ correction). D, TCGA data confirm the negative correlation between SLC35C1 and β‐catenin expression. Analysis of the TCGA database using cBioPortal revealed that the mRNA expression of SLC35C1 negatively correlates with the mRNA level of β‐catenin. mRNA level of SLC35C1 and β‐catenin was presented with log2 value

To elucidate the distribution of SLC35C1, we performed immunofluorescence and immunohistochemistry study in cells and tissues. As discussed by others,^22^ SLC35C1 locates mainly on the membrane of the Golgi apparatus. We confirmed this statement by detecting the colocalization of exogenous flag‐tagged SLC35C1 with Golgi apparatus marker protein GM130 in HEK293 cells. Meanwhile, we also noticed the colocalization of flag‐tagged SLC35C1 with calnexin, an ER marker protein, as well as Rab5 and Rab7, markers for early and late endosome. As expected, we did not find SLC35C1 in lysosome labelled by Lamp1 or mitochondria labelled by TOM20 (Figure 1). To be noted, β‐catenin was not found to be colocalizing with SLC35C1 in either healthy control tissue or cancer tissue by fluorescent immunohistochemistry (Figure 2A). Instead, multichannel colour profiling indicated that the intensity of SLC35C1 negatively correlates with β‐catenin (Figure 2B). Moreover, to investigate the relationship between SLC35C1 expression and β‐catenin level, we analysed the TCGA provisional database including 640 colorectal cancer samples with cBioPortal. Results showed that the mRNA level of SLC35C1 negatively correlates with that of β‐catenin (Figure 2C, Pearson: −.10, Spearman: −.10, *P* = .0276.).

### 3.2 SLC35C1 is down‐regulated in Colon cancer

**Figure 3:**
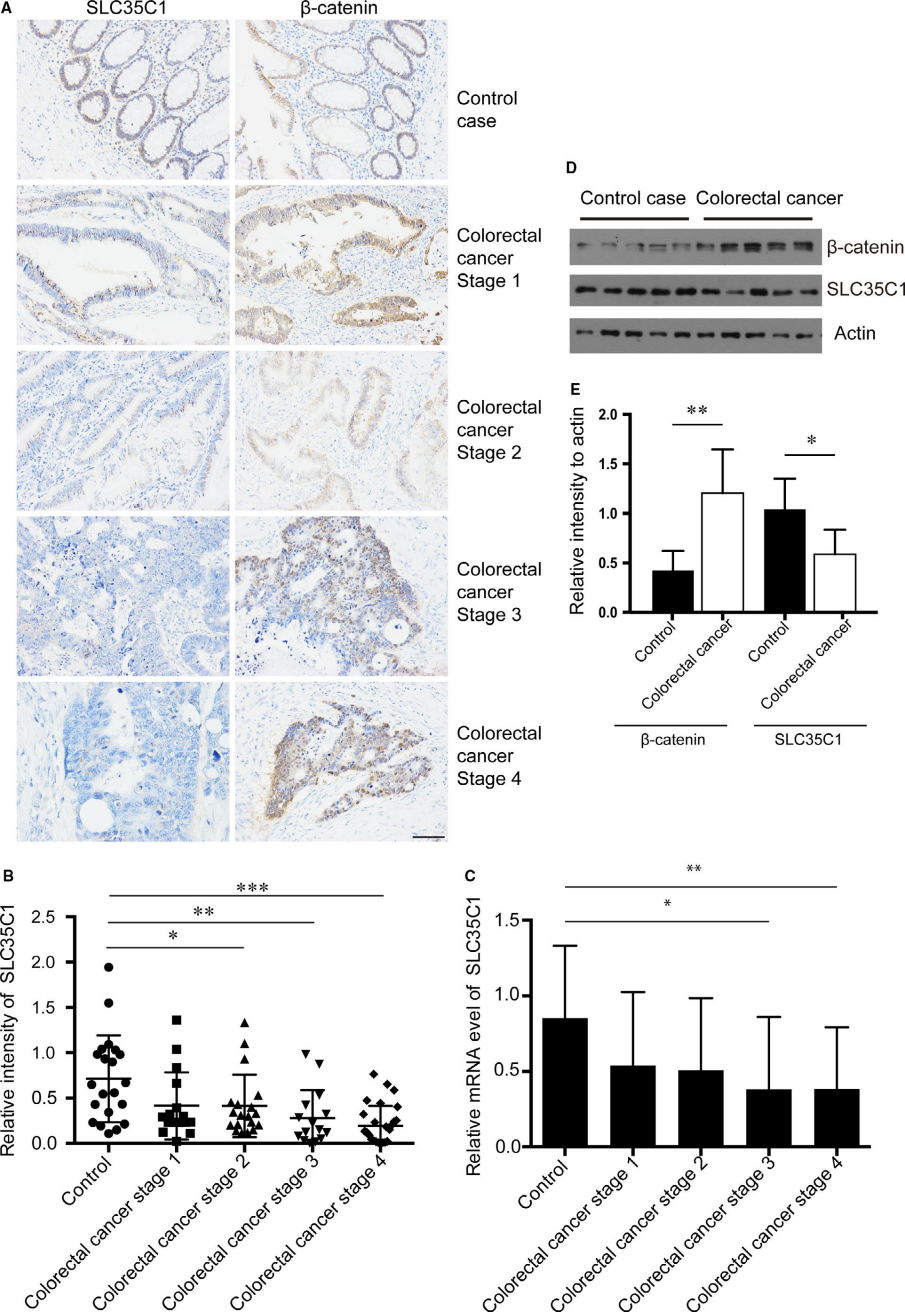
SLC35C1 is down‐regulated in colon cancer tissue. A, H&E staining shows decreased SLC35C1 and increased β‐catenin in colon cancer tissue. Tissue samples from healthy donors and colon cancer patients were subjected to immunohistochemistry assay. H&E staining revealed that SLC35C1 expression is significantly reduced at each stage of colon cancer where β‐catenin is increased. Twenty‐one healthy controls, 16 stage 1 samples, 19 stage 2 samples, 15 stage 3 samples and 24 stage 4 samples were included in the assay. B, Statistical results of Figure 1A (Mean ± SD, *P < .05, **P < .01, ***P < .001, one‐way ANOVA). The relative level of SLC35C1 was determined by calculating the average H&E intensity of each sample normalized to the average intensity of a randomly selected control sample. C, Real‐time PCR shows the mRNA level of SLC35C1 is decreased in stage 3 and stage 4 colorectal cancer. The expression of SLC35C1 is quantified by real‐time PCR and presented by the ratios between SLC35C1 and β‐actin transcripts for normalization (Mean ± SD, *P < .05, **P < .01, one‐way ANOVA). D, Western blot shows increased β‐catenin and reduced SLC35C1 level in colorectal cancer. Tissue samples were subjected to Western blot to compare the protein level of β‐catenin and SLC35C1 in healthy and colorectal cancer. β‐actin was used as the loading control. E, Statistical results of Figure 3D. (Mean ± SD, *P < .05, **P < .01, Student's *t* test.) The intensity of the bands was normalized to the intensity of matching actin bands

**Figure 4:**
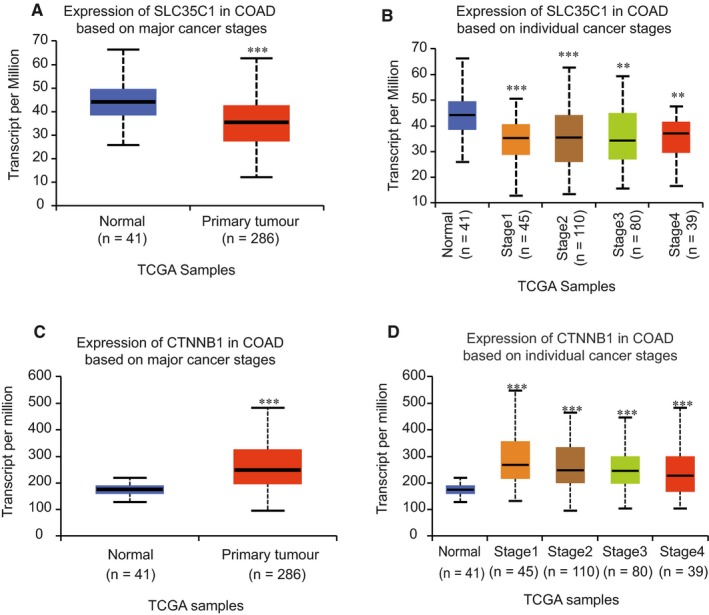
SLC35C1 is down‐regulated in colon cancer whereas β‐catenin is elevated. Colon adenocarcinoma (COAD) samples collected in TCGA base is analysed with web tool UALCAN. Results show that: A, SLC35C1 is significantly down‐regulated in primary colon cancer (*P* = .0004); B, SLC35C1 is significantly down‐regulated in all stages of colon cancer (*P* = .0002, 0.0003, 0.0057, 0.0075, respectively); C, β‐catenin is significantly down‐regulated in primary colon cancer (*P* < 1E‐12); D, β‐catenin is significantly down‐regulated in all stages of colon cancer (*P* = 9.42E‐10, *P* = 1.62E‐12, *P* = 5.52E‐14, respectively). **P < .01 and ***P < .001

Accumulating evidence suggests that abnormal activation of the canonical Wnt signalling pathway is a critical step of colon cancer development. Therefore, we wish to determine the level of SLC35C1 and β‐catenin in colon cancer tissue and control tissue. Immunohistochemistry assay revealed that SLC35C1 expression is significantly decreased in colon cancer (Figure 3A,B), whereas β‐catenin is increased (Figure 3A). This is further supported by detecting SLC35C1 mRNA level in the tissue samples using real‐time PCR, which showed that the mRNA level of SLC35C1 is reduced in stage 3 and stage 4 colorectal cancer. Consistently, analysing data collected from The Cancer Genome Atlas (TCGA) with online tool UALCAN (http:// ualcan.path.uab.edu/index.html) revealed that SLC35C1 level is decreased in primary (*P* = .0004, Figure 4A) and all four stages of colon adenocarcinoma (COAD, *P* = .0002, .0003, .0057, .0075, respectively. Figure 4B), whereas β‐catenin is up‐regulated significantly in both primary (*P* < 1E‐12, Figure 4C) and all 4 stages of COAD (*P* = 9.42E‐10, *P* = 1.62E‐12, *P* = 5.52E‐14, respectively, Figure 4D). These data strongly suggest that SLC35C1 is involved in colon cancer development.

### 3.3 Silencing SLC35C1 promotes cell growth and colony formation

**Figure 5:**
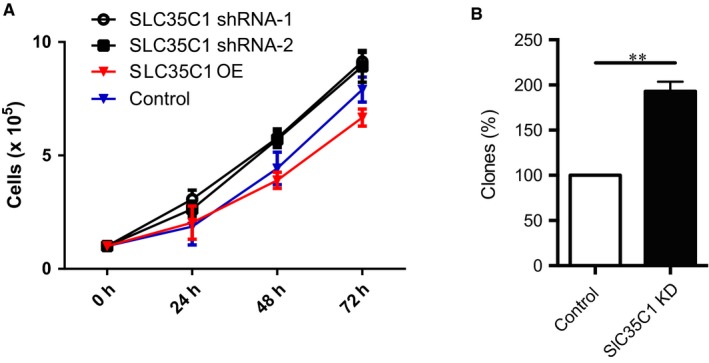
Silencing SLC35C1 promotes cell growth and clone formation. A, Control HEK293 cells and cells with SLC35C1 silenced or overexpressed were seeded in 6‐well plate. Each well started with 50 000 cells, and 4 time‐points were calculated. Each time‐point was prepared in triplicates. To calculate cells, wells were trypsinized and cell numbers were counted under a stereo microscope. Results show that cell proliferation was induced in response to SLC35C1 silencing with either shRNA constructs (*P < .05, **P < .01). Conversely, overexpressing SLC35C1 inhibits cell growth (**P < .05). SLC35C1 shRNA‐1 and SLC35C1shRNA‐2 indicate SLC35C1 silenced with two shRNA strands constructs; SLC35C1 OE indicates SLC35C1 overexpressed HEK293 cells. Data were presented as Mean ± SD. Statistic significance was analysed with two‐way ANOVA with multiple comparisons using GraphPad Prism7. B, Control HEK293 cells and cells with SLC35C1 silenced were subjected to colony formation assay. Silencing SLC35C1 significantly increased the number of colonies formed in soft agar (Mean ± SD, **P < .01)

Next, we silenced SLC35C1 in HEK293 cells and performed the growth curve assay. Comparing to control cells, decreased SLC35C1 significantly induces cell proliferation and increased cell number (Figure 5A). More interestingly, the number of HEK293 cell colonies formed in soft agar also significantly increased after silencing SLC35C1, which suggests the enhanced propagation ability when SLC35C1 level is reduced (Figure 5B). The reduction and increase of SLC35C1 expression by shRNA silencing and overexpression were confirmed by Western blot (Figure S1).

### 3.4 SLC35C1 negatively regulates the Wnt signalling pathway

**Figure 6:**
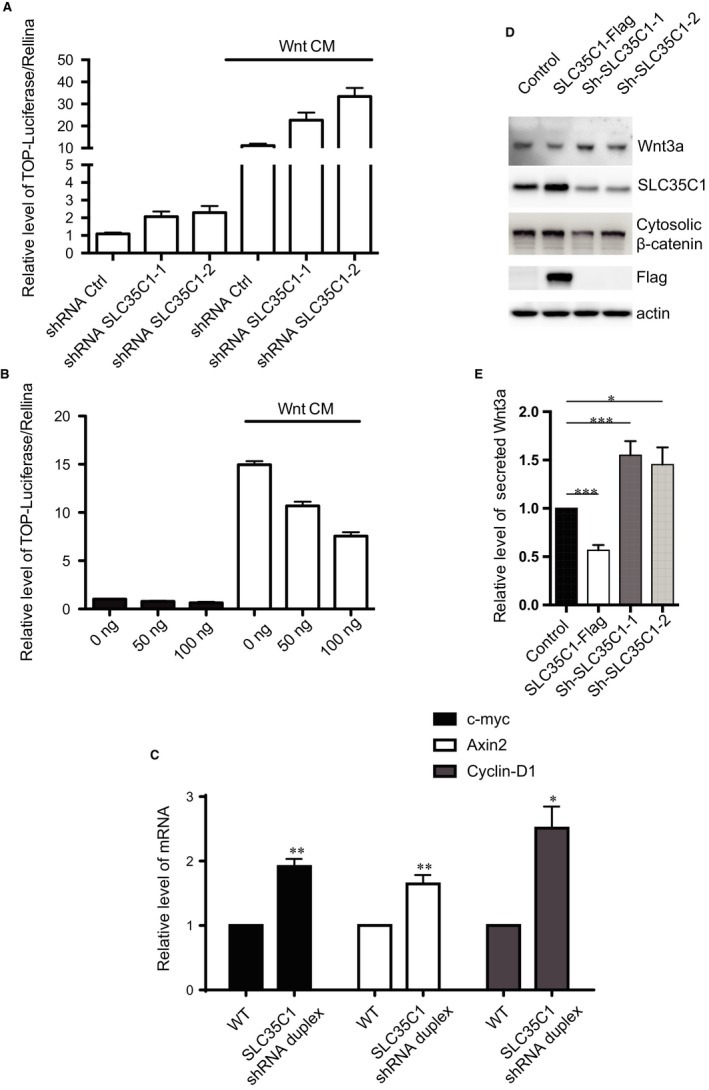
SLC35C1 negatively regulates Wnt pathway activity. A, TOPFLASH assay shows activation of the canonical Wnt pathway by silencing SLC35C1 in HEK293 cells. HEK293 cells were transfected with two different strands of shRNA. 48 h later, TOPFLASH assay was performed and revealed increased luciferase intensity after silencing SLC35C1 compared to the intensity in cells transfected with scrambled siRNA (shRNA ctrl), whereas stimulating with conditioned medium collected from L Wnt‐3A cells (Wnt CM) for 12 h significantly reversed the effect caused by SLC35C1 silence (Mean ± SD). B, TOPFLASH assay shows overexpressing SLC35C1 in HEK293 cells inhibits the canonical Wnt pathway in a concentration‐dependent manner. HEK293 cells were overexpressed with SLC35C1 (0 ng plasmid, 50 ng plasmid, 100 ng plasmid). 48 h later, TOPFLASH assay revealed decreased luciferase intensity, and this inhibition can be restored by Wnt CM stimulation for 12 h (Mean ± SD). C, Real‐time PCR shows increased mRNA level of Wnt target genes in SLC35C1 silenced HEK293 cells. Endogenous SLC35C1 gene in HEK293 cell was silenced using the shRNAs system. 48 h later, mRNA level of Wnt target genes, including C‐myc, Axin2 and Cyclin D1, was found to be elevated by real‐time PCR, suggesting that absence of SLC35C1 activates Wnt signalling pathway (Mean ± SD, *P < .05, **P < .01). D, Western blot shows silencing SLC35C1 alters the level of Wnt3a and β‐catenin in HEK293 cells. HEK293 cells were transfected with either exogenous SLC35C1 or shRNAs that silence endogenous SLC35C1. 48 h later, cells were lysed, and the culture medium was collected and centrifuged at 13 000 *g* to remove the debris. Cell lysates and centrifuged supernatants were then loaded for Western blot. Results showed that Wnt 3a level is reduced by overexpressed SLC35C1 but induced by silencing SLC35C. On the contrary, the amount of cytosolic β‐catenin is negatively associated with SLC35C1 level. E, Statistical results of Figure 6D (Mean ± SD, *P < .05, ***P < .001)

To validate the effect of the SLC35C1 level on the Wnt signalling activity, we performed the TOPFLASH assay. Comparing to the scrambled shRNA (shRNA Ctrl), both shRNA plasmids that silences SLC35C1 increased the relative luciferase signal, whereas stimulating the cells with conditioned medium collected from L Wnt‐3A cells (Wnt CM) for 12 hours augmented the effect, indicating that the reduction of SLC35C1 induced the nucleus translocation of β‐catenin and the activation of the Wnt pathway (Figure 6A). Meanwhile, overexpressing SLC35C1 decreased the luciferase intensity in an SLC35C1 level‐dependent manner (Figure 6B), even in the presence of Wnt CM, which implies that raised SLC35C1 level suppresses Wnt pathway activity. Furthermore, real‐time PCR revealed that down‐regulation of SLC35C1 by shRNA plasmids induced the mRNA level of Wnt target genes, including C‐myc, Axin2 and Cyclin D1, suggesting the activation of Wnt signalling pathway (Figure 6C). Interestingly, Western blot showed that silencing SLC35C1 in HEK293 cell results in increased secretion of Wnt3a and decreased cytosolic β‐catenin whereas overexpressing SLC35C1 have the opposite effects (Figure 6D,E). This provides evidence that one possible mechanism by which reducing SLC35C1 triggers the activation of Wnt pathway is through stimulating the secretion of the Wnt ligands. Taken together, these data indicate that down‐regulation of SLC35C1 in colon cancer induces Wnt pathway activity, whose over‐activation has been proved to be a hallmark of colon cancer.

## 4 DISCUSSION

There is intensive evidence suggesting that the canonical Wnt signalling pathway plays a critical role in the initiation and development of colon cancer.^23^, ^24^ The current study implicates SLC35C1 as a negative regulator for the canonical Wnt pathway during this process. We discovered that (a) SLC35C1 is present not only in Golgi apparatus and ER but also in early and late endosome; (b) SLC35C1 is reduced in colon cancer tissue, where β‐catenin is found increased; (c) silencing SLC35C1 in cells induces cell proliferation and enhances the colony formation ability; and (d) silencing SLC35C1 in cells induces Wnt3a secretion and activates canonical Wnt pathway. Taken together, we concluded that SLC35C1, a transporter for GDP‐L‐fucose, negatively modulates canonical Wnt pathway. Decreased SLC35C1 causes increased secretion of Wnt3a and abnormal Wnt activation, an event that is known to be the cause of tumorigenesis or metastasis.

In agreement with our findings, Ayako Kurimoto discovered that in mouse embryonic fibroblasts (MEFs) from FUT8 (ɑ‐ (1,6)‐fucosyltransferase)‐deficient mice, Wnt/β‐catenin signalling is up‐regulated, indicating fucosylation participates in the modulation of Wnt.^25^ Meanwhile, Feng et al reported that during the development of zebrafish, SLC35C1 negatively regulated Wnt signalling through N‐fucosylation of Wnt8a and Lrp6, and overexpression of SLC35C1 disrupts embryonic patterning in a transporter activity‐dependent manner. Interestingly, this group also found that at the same time, SLC35C1 expression is modulated by canonical Wnt.^26^ This suggests that the Wnt signalling pathway is a carefully regulated system and it may limit its activity via elevated expression of SLC35C1.

The secretion of Wnt ligands is known to be modulated by N‐glycosylation, and Wnt‐3a mutant that lacks N‐glycosylation was impaired with secretion.^27^ Interestingly, we found that reduced SLC35C1 promotes Wnt3a secretion, indicating fucosylation plays the opposite role of glycosylation. However, the detailed molecular mechanism of how SLC35C1 modulates the canonical Wnt signalling pathway is not discussed in the present study. We have been suggested that the reduction of the SLC35C1 level could increase the nuclear translocation of β‐catenin, which activates the signalling cascade and eventually results in the development of tumours. This is supported by the Feng group who found that strong nuclear β‐catenin was absent in the marginal blastoderm cells in GDP‐Fuc and mSlc35c1 expressing Zebrafish embryos.^26^

The Wnt signalling pathway is known to be essential for maintaining stem cell niche.^28^ To elucidate how SLC35C1 impacts intestine stem cell behaviour, our group tried to knock out *Gfr*, the ortholog of SLC35C1 in drosophila intestines. Unfortunately, we did not see any changes in the intestine development of *Gfr*‐deficient flies (data not shown). This may be due to the compensation from other GDP‐fucose transport machinery. Indeed, Ishikawa et al identified two alternative GDP‐fucose transporters in drosophila, namely *Efr and CG3774*, and the latter is a homolog of the human gene,^29^ indicating that knocking out *Gfr *alone may not be sufficient to produce the phenotype. Moreover, the fucosylation of LAD II patients can be improved by fucose supply,^22^ suggesting the existence of other GDP‐fucose transport pathways in humans.

SLC35C1 was first cloned in leucocyte adhesion deficiency II (LAD II) patients who have strongly impaired fucosylation. Fucosylation is an oligosaccharide modification that attaches fucose to proteins, lipids or other organic molecules. It is widely involved in a variety of biological processes including cell adhesion, tissue development, angiogenesis, fertilization, malignancy^30^ and cancer metastasis.^31^ Fucosylation is catalysed in Golgi or ER by glycosyltransferases and GDP‐fucose synthetic enzymes (FUTs), in the presence of the substrates and nucleotide sugars transported from the cytoplasm by GDP‐fucose transporters. Alteration of fucoprotein level and FUT activity has been reported in serum and tumours of cancer patients, including colon cancer,^32^, ^33^ lung cancer,^34^ breast cancer,^35^ gastrointestinal cancer,^36^ oral cancer,^37^ bladder cancer^38^ and endometrial cancer.^39^ To be noted, previous reports mainly focus on overexpression of fucosylated epitopes and up‐regulation of fucosyltransferases (FUTs), of which the mechanism and the impact on cancer cell biology remain unclear. For example, Villar‐Portela et al documented an increase in the level of FX enzyme and GDP‐L‐Fuc transporter in colon cancer.^40^ On the contrary, our study discovered that SLC35C1 was decreased in colon cancer.

In summary, we identified SLC35C1 as a novel negative regulator of the canonical Wnt pathway, and SLC35C1 is reduced in colon cancer. This study also offers the potential to explore drugs that can restore SLC35C1 to reduce Wnt activity and to prevent colon cancer progression.

## CONFLICT OF INTEREST

The authors declare that they have no competing interests.

## AUTHOR CONTRIBUTIONS

MZD, ZHC, and HLL acquired the funding. MZD and TJQ planned and carried out the study. HLL collected the human tissue and performed related experiments. MZD prepared the manuscript. All authors provided the final approval of the version to be published.

## ETHICAL APPROVAL AND INFORMED CONSENT

The use of human tissues was approved by the Ethics Committee of the Third Xiangya Hospital of Central South University and patients’ consents were obtained.

## Data Availability

All data are available upon request.
